# Diagnostic problems with parasitic and non-parasitic splenic cysts

**DOI:** 10.1186/1471-2482-9-9

**Published:** 2009-05-29

**Authors:** Gokhan Adas, Oguzhan Karatepe, Merih Altiok, Muharrem Battal, Omer Bender, Deniz Ozcan, Servet Karahan

**Affiliations:** 1 Okmeydani Training and Research Hospital, Department of General Surgery, Istanbul, Turkey; 2 Okmeydani Training and Research Hospital, Department of Pathology, Istanbul, Turkey

## Abstract

**Background:**

The splenic cysts constitute a very rare clinical entity. They may occur secondary to trauma or even being more seldom due to parasitic infestations, mainly caused by ecchinocccus granulosus. Literature lacks a defined concencus including the treatment plans and follow up strategies, nor long term results of the patients. In the current study, we aimed to evaluate the diagnosis, management of patients with parasitic and non-parasitic splenic cysts together with their long term follow up progresses.

**Methods:**

Twenty-four patients with splenic cysts have undergone surgery in our department over the last 9 years. Data from eighteen of the twenty-four patients were collected prospectively, while data from six were retrospectively collected. All patients were assessed in terms of age, gender, hospital stay, preoperative diagnosis, additional disease, serology, ultrasonography, computed tomography (CT), cyst recurrences and treatment.

**Results:**

In this study, the majority of patients presented with abdominal discomfort and palpable swelling in the left hypochondrium. All patients were operated on electively. The patients included 14 female and 10 male patients, with a mean age of 44.77 years (range 20–62). Splenic hydatid cysts were present in 16 patients, one of whom also had liver hydatid cysts (6.25%). Four other patients were operated on for a simple cyst (16%) two patients for an epithelial cyst, and the last two for splenic lymphangioma. Of the 16 patients diagnosed as having splenic hydatit cysts, 11 (68.7%) were correctly diagnosed. Only two of these patients were administered benzimidazole therapy pre-operatively because of the risk of multicystic disease The mean follow-up period was 64 months (6–108). There were no recurrences of splenic cysts.

**Conclusion:**

Surgeons should keep in mind the possibility of a parasitic cyst when no definitive alternative diagnosis can be made. In the treatment of splenic hydatidosis, benzimidazole therapy is not necessary, although it is crucial to perform splenectomy without rupturing and spilling the cysts.

## Background

Cystic disease of the spleen is an uncommon condition with an incidence of 0.07% in a review of over 42,000 autopsies [1]. The majority of cases are due to parasitic infection with Echinococcus granulosus resulting in hydatid disease [1,2]. Non-parasitic cysts account for less than one-third of all splenic cyst cases, and the majority of these are pseudo cysts secondary to trauma, with no epithelial lining. True non-parasitic splenic cysts make up only 10% of all benign non-parasitic splenic cysts and is rarely clinically observed [1-3]. In the literature, there are few studies on splenic cysts that show the details of diagnosis and have long term follow-up. In this study, we present the management of splenic cysts in 24 patients at our clinic and discuss the difficulties in classifying parasitic and nonparasitic cysts.

## Methods

The records of 18 out of 24 patients who underwent surgery for spleen cysts at our hospital during the period from January 1999 to December 2008 were prospectively analyzed. The records of the remaining 6 patients, who were operated by the same surgical team, were retrospectively analyzed. Then, after all the hospital records had been examined, the patients were recalled to our hospital one by one. Our exclusion criteria were multiple hydatidosis, and patients treated with percutaneous or laparoscopic management. All patients had splenectomy as the procedure of choice and the splenectomies were all performed by the same surgical team. All patients were vaccinated against Pneumococcus and Haemophilus influenzae before surgery (2 weeks prior).

The analyses focused on the differentiated diagnosis of splenic cysts and their treatment. In patients followed prospectively, the variables recorded were age, gender, cyst size, serology, mortality, morbidty and whether the disease was new or recurrent. Our retrospectively examined patients were assessed with the same parameters. An Echinococcus granulosus infection was diagnosed by imaging procedures [ultrasonography and computed tomography (CT)], serologic tests [indirect hemaglutination test (IHA)], and an enzyme-linked immunosorbent assay (ELISA).

Of the 18 prospectively observed patients, 2 received perioperative anthelmintic therapy because of their multicystic state. Each of these patients received albendazole, administered at a dose of 10 mg/kg body weight per day, from 1 week before surgery to 4 months postoperatively. During anthelmintic treatment, clinical examination and laboratory work were performed each month. The blood tests included a blood count, liver enzymes, and bilirubin assay. The other 16 patients did not undergo any antihelmitic therapy. The study plan was reviewed and approved by our institutional ethical committee, and an informed consent was obtained for all patients

## Results

**Figure 1:**
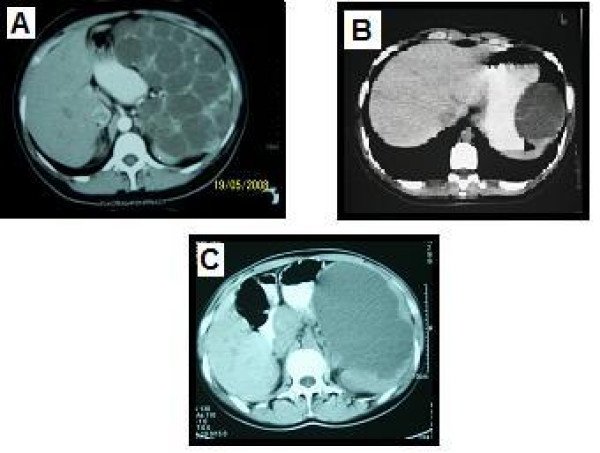
**CT view of the different cyst**. A) Cystic lenfangiomas B) Epidermoid cyst C) Cyst hidatic.

**Figure 2:**
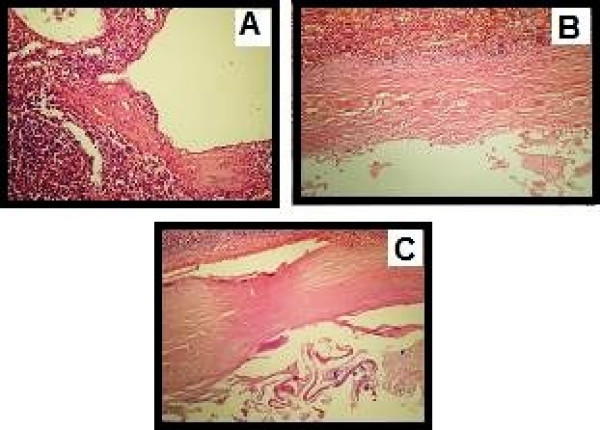
**Histopathological view of the splenic cyst**. A)cystic lenfangiomas B) psoudocyst C) cyst hydatic.

**Figure 3:**
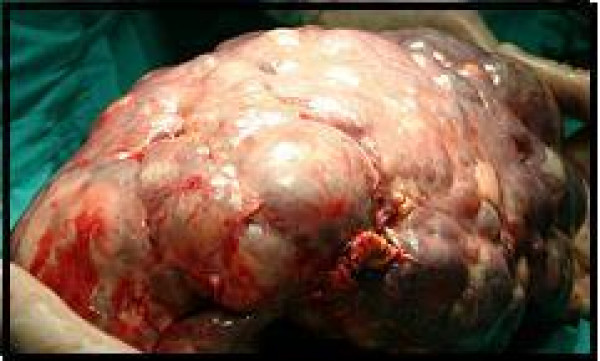
**Intraoperative view of the cystic lenfangiomas in the spleen**.

This study included 24 patients, 18 of whom were assessed prospectively, and 6 whose hospital records and treatment information were examined retrospectively. All six patients were recalled to our hospital and diagnosed as having either hydatid (4 of 6) or simple splenic cysts (the other 2). By CT and ultrasonography all 6 patients were re-examined and found to have no recurrence. The mean age of all these patients was 44.7 (range 20–62). Of the 24 patients, the number whose preoperative diagnoses was defined as parasitic 19 (79.1%), where the serologies of these patients were all positive. Four out of six patients were diagnosed as having a hydatid cyst Ultrasonography was successful in diagnosing the cystic lesions in all of them, while the serology findings were positive in only 11. A CT scan that was done in 24 patients showed a cystic lesion in all 24 (Fig. 1A, B, C). The size of the splenic cyst varied from 6 to 15 cm. The spleen was the only site of the hydatid in 14 patients (73%). An associated liver hydatid was present in 2 patients including one with renal involvement. The diagnosis of splenic parasitic cyst was confirmed in all cases by histopathology on the basis of resected specimens (Fig 2A, B, C). The most common symptoms among those operated on electively were vague abdominal pain or discomfort and/or a palpable mass in the left hypochondrium (Fig 3).

The mean hospital stay was 5.8 days with a range of 4–12 days. The mean follow-up was 64.4 months, with a range of 6 months to 108 months. None of the patients demonstrated recurrence during the follow-up. No patient had any serious septic complications. All the data are displayed in tables 1 and 2.

**Table 1:** Prospective patients detail

**Age** **n = 18**	**Gender**	**Hosp. stay**	**Cyst size**	**Additional disease**	**Serology**	**Diagnostic studies**	**Cyst recurrence**	**Follow up(month)**
50	F	6	6.6	+(liver hydatid)	+	USG	_	108
23	F	6	9	_	+	CT+USG	_	90
46	F	5	9	_	+	USG	_	85
38	F	7	8	_	+	CT+USG	_	6
52	F	6	12	_	+	CT+USG	_	14
34	F	4	16	_	+	CT+USG	_	20
49	F	6	9.5	_	_	CT+USG	_	30
62	F	12	20	_	+	CT+USG	_	45
33	F	6	10	_	+	CT+USG	_	48
38	F	5	8	_	+	CT+USG	_	75
25	M	5	9	_	+	CT+USG	_	86
28	M	5	8.5	_	+	CT+USG	_	54
43	M	7	6	_	+	CT+USG	_	76
56	M	7	10	_	_	CT+USG	_	75
60	M	9	7.5	_	_	CT+USG	_	80

**Table 2:** Retrospective patiens detail

**Age** **n = 6**	**Gender**	**Hosp. stay**	**Cyst size**	**Additional disease**	**Serology**	**Diagnostic studies**	**Cyst recurrence**
60	F	6	6.8	+(kidney hydatid)	_	USG	_
20	F	6	9	_	+	CT+USG	_
45	F	5	9	_	_	USG	_
34	F	7	8	_	_	CT+USG	_
54	M	6	12	_	_	CT+USG	_
35	M	4	16	_	_	CT+USG	_

## Discussion

Parasitic and non-parasitic cysts of the spleen have a very similar clinical presentation, but due to their differences in diagnosis and treatment, it is important to distinguish the two. The majority of splenic cysts, both parasitic and non-parasitic, are found incidentally on imaging [4]. Splenic cysts grow slowly and are characterized by a long period of asymptomatic latency, such that initial diagnosis is usually established during the course of investigations carried out for other diseases. When the cyst reaches a considerable size, its clinical presentation is normally a result of pressure on adjacent viscera, and may include renal arterial compression with systemic hypertension, rupture to other organs, spontaneous cutaneous fistulization, or segmental portal hypertension. However, mild discomfort and pain in the left upper quadrant are the most common complaints [5]. Splenic hydatidosis can be diagnosed preoperatively with radiologic studies [4]. However, sonographic and CT findings of splenic hydatidosis are not specific, and other splenic cystic lesions, such as an epidermoid cyst, splenic abscess, a pseudocyst, or a cystic neoplasm of the spleen, may present with a similar appearance. The differential diagnosis is greatly helped by the history, the presence of calcification in the cyst wall, the presence of daughter cysts in a large cystic lesion or concominant cystic lesions in the liver or other organs [5,6]. In patients with negative serology and indeterminant US and CT examinations, it may be helpful to use MRI. A recent article [6] has shown the value of MRI in differentiating parasitic, nonparasitic, traumatic or other unilocular cyst by demonstrating: "A low-signal intensity rim so-called rim sign, can be seen on both T1 & T2 weighted MR images but more conspicuous or evident on T2 weighted images, has been described as characteristic of hydatidosis as opposed to nonparasitic cysts in the liver and lungs. The low intensity rim represents the fibrous collagenous pericyst with or without calcifications. However, this sign is a nonspecific sign or finding as it can be also seen in amebic abscess, hematoma, and hepatocellular carcinoma of the liver. Furthermore, the newer applications of functional MRI (MR Diffusion & MR Spectroscopy) can help to differentiate between parasitic versus non-parasitic splenic cysts"[6].

In this study, there were no problems observed in patients preoperatively diagnosed clearly as parasitic or non parasitic. The major problem was with those cases that could not be differentiated as parasitic or non-parasitic by imaging and the serologies negative. There were no differences between labarotory findings and CT or USG images in these cases. The diagnoses were made intraoperatively or postoperatively by pathological examination. All postoperatively-diagnosed patients had hydatid cysts belonging to Gharbi level I (Fig 2A, B, C).

As seen here, diagnostic confusion was related to the type of the hydatid cysts. In the clinical setting, the pre-operative diagnosis of splenic cysts is of significance because the treatment of splenic cysts has changed over the years to a more conservative approach [6-10]. Due to the increased use of laparoscopic techniques, the recurrence of hydatidic disease resulting from spillage of cystic fluid during carelessly-performed operations may increase [8]. The classic approach to splenic cysts has been total splenectomy. However, the risk of overwhelming postsplenectomy sepsis has led to the development of paranchyma-preserving surgical procedures [11]. In recent years, percutaneous drainage of the splenic hydatid cyst with injection and consecutive reaspiration of a scolocidal agent (PAIR technique) has been proposed as an alternative, non-surgical therapy for patients at high anesthetic risk or who do not consent to surgery [5]. The high risk of septic complications caused the major change in the approach to spleen surgery.

The basic measures to prevent the recurrence of hydatid cysts include the reduction of their viability by using drugs, the prevention of cystic fluid spillage and the complete removal of their germinative membranes, which is achieved by splenectomy [12,13]. Benzimidazole carbamates (mebandazole and albendazole) are antihelminthic drugs that kill the parasite by impairing its glucose uptake [14-16]. Although albendazole reaches a high blood concentration after oral administration, cystic fluid concentration of the drug is low and clinical outcome is unpredictable [16,17]. The efficacy of preoperative albendazole treatment was established by Wen et al [18]. Preoperative treatment with a benzimidazole drug should begin at least four days before surgery (WHO Informal Working Group on Echinococcosis,1996). In current practice, indications for benzimidazole therapy used in the treatment of hydatid disease are inoperable primary hepatic hydatic hydatidosis, multiple cysts in two or more organs, multiple small liver cysts, cysts located deep in the hepatic parenchyma, prevention and management of secondary hydatidosis, management of recurrent hydatidosis, unilocular cysts in unfit elderly patients, adjuvant therapy with surgery or percutaneous interventions, pulmonary echinococcosis, and long-term treatment for cystic echinococcosis in specific organs like the bone, brain, or eye [19]. Four different goals can be pursued with medical management: definite cure, reduction of cyst viability, preoperative treatment, and perioperative prophylaxis. Definitive cure of univesicular cysts requires a 3- to 6-month course of medication. Reduction in the viability of the cysts can be achieved in multivesicular cysts and preoperatively in univesicular cysts when percutaneous or elective surgery is planned [14]. There are only a few papers about the preoperative or perioperative sterilization of hydatid cysts [20]. However, the reported evidence suggests that these drugs act principally parasitostatically and not as parasiticides because the disease recurs when the drugs are discontinued [21]. This means that they cannot cure the patient. However, there are case reports regarding possible parasiticidal effects following long term chemotherapy. Hence, the real efficacy of the benzimidazoles have not been entirely determined [22]. Moreover, Monterola et al. reported that the preoperative regimen for sterilising cysts was only 40% effective. They also reported that there was no association between the concentration of albendazole in the hydatid fluid and viability of the scolices [23].

With respect to splenic hydatic cysts, Ahmad et al. maintained that splenectomy remains the procedure of choice in cases of splenic hydatidosis and that benzimidazole therapy has been proven to be effective in preventing the recurrence of hydatid disease [24]. A point that needs to be discussed here is whether postoperative anti-parasitic treatment is required. In the literature, there are few studies concerning this issue [25-28]. Although not proven scientifically, benzimidizole therapy for the treatment of splenic cysts is still in practice. In our study we did not use benzimidizole therapy in any of our cases and did not confront any recurrences in the mean seven-year follow-up. We chose not to use this drug because we believed we had completely eradicated the hydatid cysts using splenectomy. Now, there is a question that comes to mind: Should postoperative anti-parasitic treatment be routinely used even after splenectomy because of hydatid disease? This question needs further prospective, randomized studies. Our results show that if the surgeon totally excises the splenic cyst, and there is no spillage into operative area, it is possible to avoid use of benzimidazole therapy after the operation.

## Conclusion

Despite the advances in medical technology, it continues to be hard to differentiate between parasitic and non-parasitic splenic cysts. Patients in whom this differentiation cannot be made should be considered to have a parasitic cyst and the operation should proceed accordingly. Even though the efficacy of benzimidazole in splenic hydatid has not been proven, it is an option depending on intraoperative conditions. Therefore, the surgeon should keep in mind the probability of a parasitic cyst when no differentiation of splenic cysts can be made. In addition, in the treatment of the splenic hydatidosis, benzimidazole therapy may or may not be used, but it is vital that splenectomy is performed without any spillage of the cyst contents.

## Competing interests

The authors declare that they have no competing interests.

## Authors' contributions

GA: Participated in its design and checked revision. OK: Write a manuscript and participated in its design, coordination. MA: Patients follow up and data pick up. MB: Patients follow up. OB: Patients follow up. DO: Pathological examination. SK: analysis and interpretation of data.

## Pre-publication history

The pre-publication history for this paper can be accessed here:


